# Improving Imaging of the Brainstem and Cerebellum in Autistic Children: Transformation-Based High-Resolution Diffusion MRI (TiDi-Fused) in the Human Brainstem

**DOI:** 10.3389/fnint.2022.804743

**Published:** 2022-03-03

**Authors:** Jose Guerrero-Gonzalez, Olivia Surgent, Nagesh Adluru, Gregory R. Kirk, Douglas C. Dean III, Steven R. Kecskemeti, Andrew L. Alexander, Brittany G. Travers

**Affiliations:** ^1^Waisman Center, University of Wisconsin-Madison, Madison, WI, United States; ^2^Department of Medical Physics, University of Wisconsin-Madison, Madison, WI, United States; ^3^Neuroscience Training Program, University of Wisconsin-Madison, Madison, WI, United States; ^4^Department of Radiology, University of Wisconsin-Madison, Madison, WI, United States; ^5^Department of Pediatrics, University of Wisconsin-Madison, Madison, WI, United States; ^6^Department of Psychiatry, University of Wisconsin-Madison, Madison, WI, United States; ^7^Occupational Therapy Program in the Department of Kinesiology, University of Wisconsin-Madison, Madison, WI, United States

**Keywords:** dMRI (diffusion magnetic resonance imaging), MPnRAGE, autism, brainstem, boundary-based registration

## Abstract

Diffusion-weighted magnetic resonance imaging (dMRI) of the brainstem is technically challenging, especially in young autistic children as nearby tissue-air interfaces and motion (voluntary and physiological) can lead to artifacts. This limits the availability of high-resolution images, which are desirable for improving the ability to study brainstem structures. Furthermore, inherently low signal-to-noise ratios, geometric distortions, and sensitivity to motion not related to molecular diffusion have resulted in limited techniques for high-resolution data acquisition compared to other modalities such as T1-weighted imaging. Here, we implement a method for achieving increased apparent spatial resolution in pediatric dMRI that hinges on accurate geometric distortion correction and on high fidelity within subject image registration between dMRI and magnetization prepared rapid acquisition gradient echo (MPnRAGE) images. We call this post-processing pipeline T1 weighted-diffusion fused, or “TiDi-Fused”. Data used in this work consists of dMRI data (2.4 mm resolution, corrected using FSL’s Topup) and T1-weighted (T1w) MPnRAGE anatomical data (1 mm resolution) acquired from 128 autistic and non-autistic children (ages 6–10 years old). Accurate correction of geometric distortion permitted for a further increase in apparent resolution of the dMRI scan via boundary-based registration to the MPnRAGE T1w. Estimation of fiber orientation distributions and further analyses were carried out in the T1w space. Data processed with the TiDi-Fused method were qualitatively and quantitatively compared to data processed with conventional dMRI processing methods. Results show the advantages of the TiDi-Fused pipeline including sharper brainstem gray-white matter tissue contrast, improved inter-subject spatial alignment for group analyses of dMRI based measures, accurate spatial alignment with histology-based imaging of the brainstem, reduced variability in brainstem-cerebellar white matter tracts, and more robust biologically plausible relationships between age and brainstem-cerebellar white matter tracts. Overall, this work identifies a promising pipeline for achieving high-resolution imaging of brainstem structures in pediatric and clinical populations who may not be able to endure long scan times. This pipeline may serve as a gateway for feasibly elucidating brainstem contributions to autism and other conditions.

## Introduction

Precise quantification of brainstem microstructure in autistic^1^ children is important as cytoarchitectural properties of the brainstem may contribute to the etiology of autism spectrum disorder (ASD). Brainstem white matter in autistic youth has been associated with motor skills (Hanaie et al., 2013; Travers et al., 2015; Surgent et al., 2021), sensory features (Jou et al., 2009; Wolff et al., 2017), and core autism traits (Travers et al., 2015; Wolff et al., 2017). Further, epidemiological, behavioral, histological, and model organism-based studies have generated hypotheses regarding brainstem contributions to ASD [reviewed by Dadalko and Travers (2018)]. Intriguingly, the first biology-based theory of autism (Rimland, 1964) suggested that autism traits were associated with abnormalities in the reticular formation, a cluster of gray matter nuclei within the brainstem. However, direct testing of this hypothesis has been limited by technical challenges that have prevented high-resolution imaging capable of probing the detailed structures of the brainstem *in vivo*.

Traditional magnetic resonance imaging (MRI) has lacked sufficient quality to characterize the intricately interwoven white matter bundles that wrap around the non-uniformly shaped gray matter nuclei within the brainstem, which itself is a relatively small structure. Structural, T1-weighted (T1w) MRI can achieve high spatial resolution but demonstrates poor contrast between the gray and white matter in the brainstem. This poor contrast makes it challenging to distinguish specific brainstem white matter tracts and gray matter nuclei. In comparison, diffusion MRI (dMRI), a powerful neuroimaging modality for *in vivo* quantification of white matter microstructure, can distinguish between different tissue types and fiber orientations within the brainstem, thereby revealing distinctions among brainstem substructures. However, geometric distortions that impact the brainstem are common in dMRI (Jezzard and Balaban, 1995; Du et al., 2002; Irfanoglu et al., 2015) and can make brainstem white matter tracts appear spuriously intertwined (Irfanoglu et al., 2012). Additionally, higher dMRI spatial resolution is needed due to the small size of brainstem structures (Ford et al., 2013; Lützkendorf et al., 2018) but comes at the cost of a lower signal-to-noise ratio (SNR) at each voxel (Edelstein et al., 1986; Jones, 2012), much longer scan times, or amplified imaging artifacts due to bulk motion and magnetic field inhomogeneities (Holdsworth et al., 2019). Moreover, increased involuntary head motion in autistic children (Yendiki et al., 2014) as well as physiological motion related to cerebrospinal fluid (CSF) pulsation (Karampinos et al., 2009), is likely to exacerbate these limitations, making imaging of brainstem structures even more challenging in autistic individuals.

**Figure 1:**
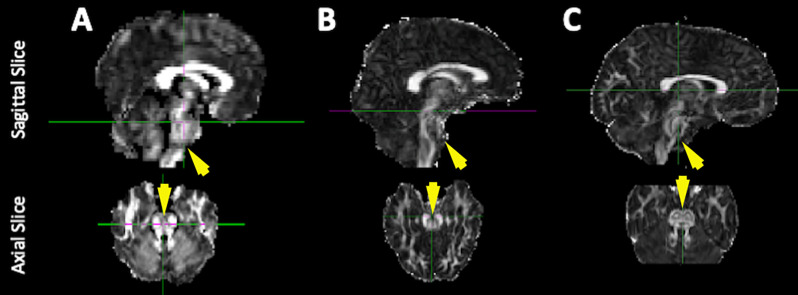
Improvements to the quality of brainstem imaging through modifications to dMRI acquisition. Representative diffusion-weighted images from our previous work including the current conventional method. Example FA maps derived from dMRI scans **(A)** with low spatial resolution and no EPI distortion correction [used in Travers et al. (2015)], **(B)** with higher spatial resolution but no EPI distortion correction (collected between 2014 and 2016), and **(C)** with higher spatial resolution and EPI distortion correction (collected between 2016 and 2020, conventional processing pipeline).

To address these dMRI challenges, we recently implemented a dMRI protocol that improves brainstem images by using multi-shell diffusion acquisition to adjudicate among crossing fibers (Pines et al., 2020) and by correcting for brainstem-impacting echo planar imaging (EPI) geometric distortions using multiple non-diffusion-weighted volumes with reverse phase-encoded directions (Andersson et al., 2003; Smith et al., 2004). Using these dMRI images and what we will here forth refer to as the “conventional” dMRI processing pipeline, we found improved delineations among the white matter tracts of the brainstem compared to previous dMRI processing pipelines (Figure 1). However, further improvements to the apparent spatial resolution of brainstem dMRI at the acquisition level would require increased scan time and/or decreased SNR, neither of which are viable options. Longer scan times would not be feasible for our sample of autistic children and decreased SNR would have negative cascading impacts on dMRI scan quality. Therefore, to address the need for higher apparent spatial resolution in these data, the present study tests a pipeline for combining (or fusing) T1w and dMRI scans [**T**1 weighted-diffusion **fused**, or “TiDi-Fused” for short (phonetically pronounced *tai-dee*)] that ameliorates common challenges in pediatric brainstem imaging and enables increased apparent resolution of the brainstem and cerebellar structures in autistic and non-autistic children. This technique combines the complementary strengths of both the T1w and dMRI scans to enhance tissue contrast and apparent spatial resolution in the brainstem and surrounding regions. To address the limitation of head motion in the TiDi-Fused processing, our T1w images were acquired with magnetization prepared rapid acquisition gradient echo (MPnRAGE; Kecskemeti et al., 2016, 2018; Kecskemeti and Alexander, 2020a). MPnRAGE retrospectively addresses head motion artifacts that are common in pediatric neuroimaging (Kecskemeti and Alexander, 2020b), provides more reproducible cortical region definitions (Kecskemeti et al., 2021), and produces sharper delineations of gray/white matter boundaries than standard T1w images (Kecskemeti et al., 2018). The anatomical accuracy of MPnRAGE allows for high fidelity dMRI-to-T1w boundary-based registration.

Therefore, the aim of the present study was to compare the quality of our TiDi Fused post-processing method with our conventional dMRI method in autistic and non-autistic children (ages 6–10 years) using the following techniques: (1) visual comparison of image characteristics in relation to histology-based atlases (Sitek et al., 2019); (2) region-of-interest-based comparisons of coefficients of variation (CoV) in an atlas of brainstem-cerebellar white matter tracts (Tang et al., 2018); and (3) effect size comparisons of age predictions for the brainstem-cerebellar white matter tracts. We hypothesized that the TiDi-Fused brainstem images would show both visual and quantitative improvements over the conventional pipeline through the visibly clearer brainstem and cerebellar structural distinctions, improved registration with histologically derived brainstem atlases, lower CoVs (reduced variability) across brainstem tracts of interest, and stronger, positive relationships between age and apparent fiber density (AFD; Raffelt et al., 2012b).

## Materials and Methods

### Participants

One-hundred and twenty-eight participants (ages 6.0–10.97, 37 female) were included in this study, with 56 in the autistic group (6.14–10.84 years, 12 females) and 72 in the non-autistic group (6.02–10.97 years, 25 females). No participants had a previous diagnosis of tuberous sclerosis, Down syndrome, fragile X, hypoxia-ischemia, notable and uncorrected hearing or vision loss, or a history of severe head injury. The institutional review board at the University of Wisconsin-Madison approved all procedures. In each case, the child participant provided assent, and a parent or guardian provided informed consent.

To confirm previous community diagnoses of ASD, participants in the autistic group were comprehensively evaluated and met cutoffs on either: (1) the Autism Diagnostic Observation Schedule, 2nd edition (ADOS-2; cutoff = 8; Lord et al., 2012) and the Autism Diagnostic Interview-Revised (ADI-R; Rutter et al., 2003b) or (2) the Social Responsiveness Scale, second edition (SRS-2; cutoff = 60; Constantino and Gruber, 2012) and the Social Communication Questionnaire (SCQ; cutoff = 15; Rutter et al., 2003a). Non-autistic participants were required to score less than eight on the SCQ (Rutter et al., 2003a). Additionally, participants were excluded from the non-autistic group if they had a previous diagnosis of another neurodevelopmental disorder, including ADD/ADHD, bipolar disorder, major depressive disorder, or if they had a first-degree relative with ASD. Supplementary Table 1 summarizes participant details.

### Image Acquisition

Imaging data were acquired on a 3T GE Discovery MR750 scanner (Waukesha, WI) at the Waisman Center at the University of Wisconsin–Madison. Diffusion-weighted images (DWIs) were obtained using a 32-channel phased array head coil (Nova Medical, Wilmington, MA) and a multi-shell spin-EPI pulse sequence [9 directions at *b* = 350 s · mm^−2^, 18 directions at 800 s · mm^−2^, and 36 directions at *b* = 2,000 s · mm^−2^, and 6 non-diffusion-weighted (*b* = 0 s · mm^−2^) volumes; TR/TE = 9,000/74.4 ms; FOV = 230 mm × 230 mm, in-plane resolution 2.4 mm × 2.4 mm, interpolated with zero-filling to 1.8 mm × 1.8 mm; 76 slices, slice thickness 3.6 mm, slice spacing 1.8 mm; ~10 min]. An additional six non-diffusion-weighted volumes with reverse phase-encoded direction were collected for use in correcting susceptibility-induced artifacts, which tend to be severe around the brainstem in EPI acquisitions. Whole-brain structural imaging was done using a 3D T1w MPnRAGE sequence with 1 mm isotropic resolution (~8 min). The MPnRAGE pulse sequence is a novel imaging method that combines magnetization preparation using inversion recovery with a rapid 3D radial k-space readout (Kecskemeti et al., 2016). The MPnRAGE reconstruction enables retrospective head-motion correction (Kecskemeti et al., 2018), tissue-specific segmentation, and reliable quantitative T1 mapping (Kecskemeti et al., 2021).

### Image Processing

#### Data Curation

A comparison of the conventional and TiDi-Fused pipelines are summarized in Table 1. In both pipelines, DWI data were processed to minimize noise (Veraart et al., 2016a,b), Gibbs ringing (Kellner et al., 2016), motion, eddy current (Andersson and Sotiropoulos, 2016; Andersson et al., 2016, 2017) and EPI distortion artifacts (Andersson et al., 2003).

**Table 1:** Comparison of conventional and TiDi-Fused processing pipelines.

	Conventional pipeline	TiDi-Fused pipeline
DWI Acquisition	
Pulse Sequence	Multi-shell spin EPI pulse sequence	Multi-shell spin EPI pulse sequence
Resolution	In-plane resolution 2.4 × 2.4 mm, interpolated to 1.8 × 1.8 mm	In-plane resolution 2.4 × 2.4 mm, interpolated to 1.8 × 1.8 mm.
Data Curation	Denoising, corrections for Gibbs Ringing, eddy currents, EPI distortion	Denoising, corrections for Gibbs Ringing, eddy currents, EPI distortion.
Apparent Resolution Enhancement*	Upsample DWI to achieve apparent resolution of 1.3 mm	Fuse T1-weighted and diffusion images with boundary-based registration (BBR) to achieve apparent resolution of 1.0 mm.
Diffusion Data Modeling	Estimate FOD and apparent fiber density (AFD)	Estimate FOD and apparent fiber density (AFD)
Population Template Construction*	Construct FOD population template using MRTrix3	Construct T1-weighted population template using ANTs.
Inter-Subject Spatial Normalization*	Diffeomorphically transform individual FOD maps to FOD template	(1) Diffeomorphically transform individual T1-weighted images to T1-weighted population template (2) Apply transformations to individual FOD maps.
Atlas Alignment*	Align FOD template to atlas (MNI) space using ANTs	Align T1-weighted template to atlas (MNI) space using ANTs.
Region of Interest Mapping to Individual Native Space	Transform data using warps generated from inter-subject spatial normalization	Transform data using warps generated from inter-subject spatial normalization.
Apparent Fiber Density (AFD) Value Extraction	Calculate the weighted median values from regions/tracts of interest in individual native space	Calculate the weighted median values from regions/tracts of interest in individual native space.

*Denotes steps in which the method pipelines differ.

#### Apparent Spatial Resolution Enhancement

In the conventional pipeline, a spatial resolution of dMRI data was up-sampled using MRtrix3’s “mrgrid” (Tournier et al., 2019) with cubic interpolation to 1.3 mm isotropic voxels prior to estimating the fiber orientation distributions (FODs^2^).

In the TiDi-Fused pipeline, image-modality fusion was used to enhance the apparent spatial resolution. Image-modality fusion was conducted *via* spatial alignment of mean b0 volume to the T1w image derived from the MPnRAGE. The spatial alignment was done using rigid transformations (six degrees of freedom) implemented with the boundary-based registration (BBR; Greve and Fischl, 2009) routine in the FreeSurfer image analysis suite (Dale et al., 1999). With BBR, brain tissue boundaries estimated with FreeSurfer on the T1w image were maximally aligned with the expected image intensity gradients across those boundaries in the b0 image. The estimated transformation that resulted from the optimal alignment was then applied to the entire dMRI series with cubic B-spline interpolation up-sampled to the T1-w resolution (1 mm isotropic) using ANTs (Avants et al., 2011). Finally, the rotational component of the rigid body transformation was applied to the dMRI encoding directions. Subsequently, multiple operations on the diffusion scans, including estimation of fiber orientation distributions, were carried out in the up-sampled space.

#### Fiber Orientation Distribution

In the conventional and TiDi-Fused pipelines, dMRI data were spherically deconvolved with positivity constraints (Jeurissen et al., 2014) using an estimated shell and tissue specific response functions (Dhollander et al., 2016; averaged across the participants in the study) in order to estimate fiber orientation distributions (FODs) at each voxel in the brain as shown in Table 1.

#### Apparent Fiber Density

In both pipelines, following FOD estimation, apparent fiber density (AFD; Raffelt et al., 2012b) was calculated. AFD represents the sum of the amplitudes of the FOD lobes in each voxel and has been proposed as a measure of intracellular volume fraction from high angular resolution dMRI (Raffelt et al., 2012b). In order to generate AFD, global intensity normalization in the log-domain was first performed on the three different (WM, GM, CSF) FODs using the “mtnormalise” command in MRTrix3 (Dhollander et al., 2021). With normalized white matter FOD maps in hand, the apparent fiber density was computed as the first component (*l* = 0; typically referred to as DC term) of the white matter FOD series scaled by 2√π.

#### Inter-subject Image Alignment

In the conventional pipeline, an FOD template was created using the “population_template” routine in MRtrix3 with default settings (Tournier et al., 2019). This template was estimated from all 128 individuals, thus resulting in spatially aligned individual FOD maps to the template and corresponding diffeomorphic and affine transforms.

In the TiDi-fused pipeline, a study-specific T1-w template was first estimated using the “antsMultivariateTemplateConstruction” script in ANTs with four iterations (Avants et al., 2010, 2011; Klein et al., 2010). This template was also estimated from all participants. The resulting affine and local non-linear transformations were composed and then applied to the FOD maps with FOD reorientation using MRTrix3 (Raffelt et al., 2012a). An FOD template was then created from the aligned FOD maps by averaging them across subjects. This resulted in the T1w and FOD templates in the same spatial coordinate system.

#### Histological and Probabilistic Atlas Registration

Apparent fiber density was examined in 23 brainstem and cerebellar probabilistic white matter tracts defined in an atlas in which the tracts were identified by filtering whole brain tractography using regions manually defined by a neuroanatomist as described in more detail in Tang et al. (2018). The MNI152 template was aligned to the FOD templates created by the two processing pipelines. In the conventional pipeline, this was achieved by affine and diffeomorphic image alignment between the DC term of the template FOD and the MNI T1w image using “antsRegistration” (Avants et al., 2011) and mutual information as the cost function in the non-linear stage. In the TiDi-Fused pipeline, the MPnRAGE-T1w study-specific template was aligned with the MNI152 T1w image also using “antsRegistration” with affine and diffeomorphic transformations with correlation coefficient as the cost function in the non-linear stage. The probabilistic tract atlas was transformed to the FOD template space using the estimated warps and cubic interpolation. The tracts were then mapped to subject specific native space by applying the inverse transformations estimated during the template construction.

Additionally, histology-based data were aligned to the FOD templates created in each pipeline using the same procedure outlined above. The data consist of the BigBrain 3-D Volume Data Release 2015 (Amunts et al., 2013) 100 μm version with optimal alignment to ICBM152 2009b non-linear symmetric^3^, 500 μm (Fonov et al., 2009) T1w template conducted by Sitek et al. (2019). The auditory brainstem nuclei also published in Sitek et al. (2019), were then mapped to each of the estimated FOD templates. A comparison of the histological data aligned to ICBM152 and the study specific template spaces is shown in Supplementary Figure 1.

#### Track Density Imaging

**Figure 2:**
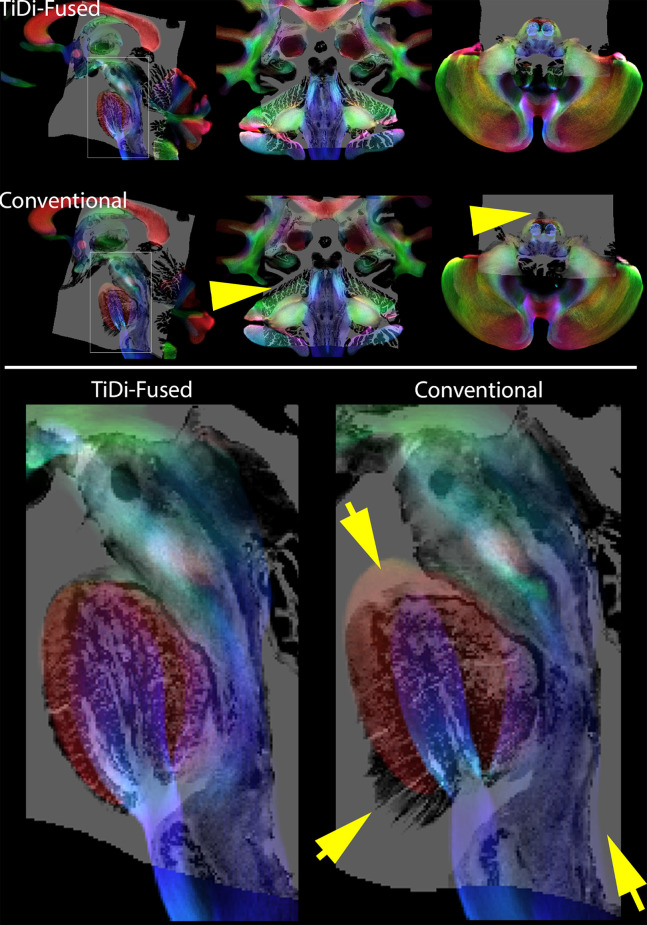
Histology overlays on TDI. Top panel: Histology on Track Density Imaging maps resulting from each of the two pipelines—sagittal (right), coronal (middle), and axial (left). Bottom panel: Amplified sagittal view of histology on TDI in brainstem highlights the better spatial alignment resulting from TiDi-Fused. Note the arrows pointing to areas were the conventional pipeline leads to badly aligned regions with histology. In contrast, using TiDi-Fused leads to a mapping of histology that is much better supported by the underlying TDI contrast.

Track density imaging (TDI) is a post processing approach based on tractography that offers the ability to increase anatomical contrast in white mater (Calamante et al., 2010). To visually compare the resulting contrast in TDI in the brainstem with the BigBrain histology data, we produced TDI maps based on the FOD templates generated with each of the two pipelines. In each case, whole brain probabilistic tractography was performed using MRTrix3 using the white matter FODs seeded from a white matter mask that was generated by thresholding the DC term (≥0.05) of the FOD (Tournier et al., 2010). Twenty million streamlines were generated and used to calculate TDI maps at an isotropic spatial resolution of 0.25 mm. To display directional information of fibers, TDI maps were represented as directionally encoded color maps. Histology overlaid on the TDI maps is shown in Figure 2.

### Statistical Analysis

Weighted median values of the AFD in 23 brainstem tracts in native space were extracted for both the conventional dMRI and TiDi-Fused pipelines. Coefficients of variation (CoVs) across the subjects defined as the ratio of the standard deviation to the mean values of the weighted medians, were computed for each of the tracts. Statistical differences between the two pipelines were assessed using a Wilcoxon signed-rank test, in consideration that the data may not be normally distributed.

Pearson correlations between age and AFD in the conventional and TiDi-Fused pipelines were performed for each brainstem white matter region of interest. Model fit was tested through examination of R^2^ values to represent explained variance. To directly test whether there were significant differences in the model fit between the conventional and TiDi-Fused pipelines, a mixed effects linear model was also conducted in each region of interest, predicting AFD as a function of age, pipeline (conventional vs. TiDi-Fused), and the age-by-pipeline interaction.

## Results

### Enhanced Brainstem Visualization

**Figure 3:**
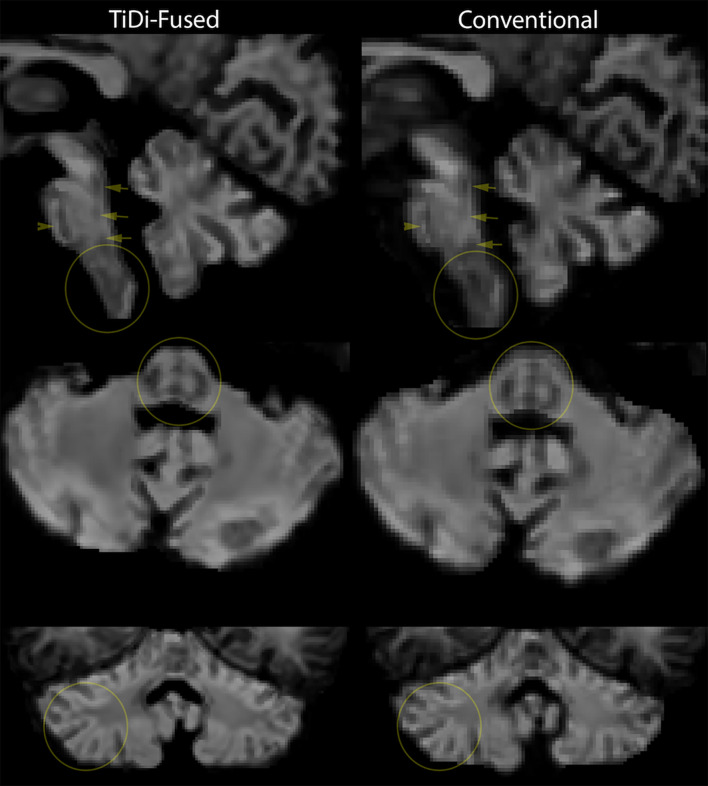
Single-subject-level improvement (non-autistic 7-year-old) in gray-white matter contrast seen in apparent fiber density (AFD) in the brainstem and cerebellum. We chose the first scan of the study to demonstrate this, but this effect was representative of the dataset.

TiDi-Fused processing of dMRI images resulted in enhanced visualization of gray and white matter structures within the brainstem and cerebellar areas compared to conventional dMRI processing. Figure 2 visually shows the benefits of the TiDi-Fused pipeline in terms of spatial alignment of population-level data to a histologically derived atlas of the brainstem. Specifically, TiDi-Fused processed images show crisp alignment with histologically defined boundaries in the pontine region and strong registration of dorsal brainstem white matter tracts, demonstrating the effects of enhanced apparent resolution and improved tissue contrast in the TiDi-Fused images. At the single subject-level, dMRI images processed with the TiDi-Fused pipeline, demonstrate improved visibility of gray-white matter boundaries and sharpened patterns of cerebellar arborization (Figure 3).

### Improved Precision in Estimates of White Matter Properties

**Figure 4:**
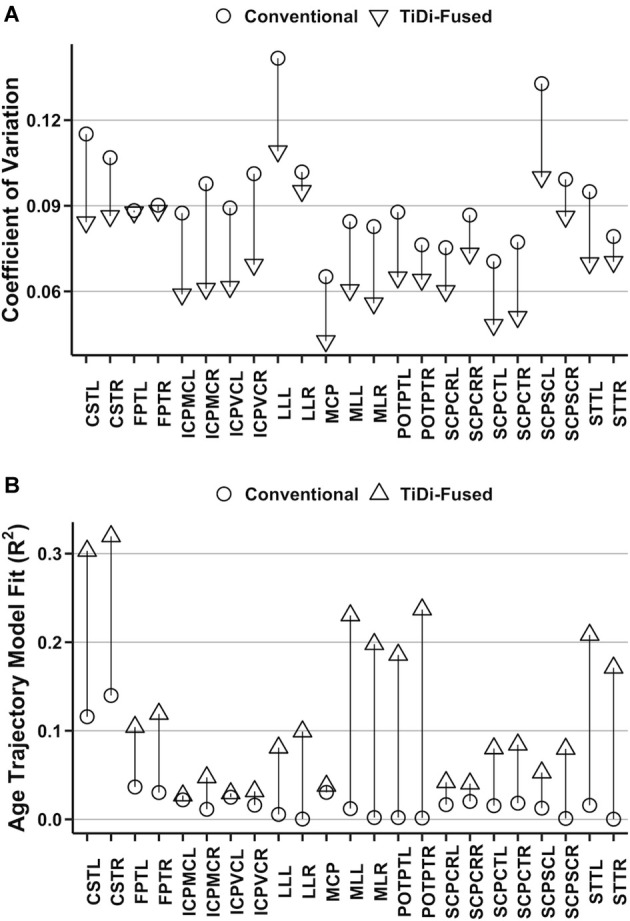
Quantitative analysis of original and optimized pipelines using brainstem white matter regions of interest. **(A)** Comparison of coefficient of variation in the original and optimized pipelines. **(B)** Proportion of age trajectory variance (R^2^) explained by apparent fiber density (AFD) in each white matter tract.

Improvements to estimates of brainstem white matter microstructural properties were assessed through analysis of AFD CoV within 23 brainstem white matter tracts that were precisely delineated in dMRI data processed with conventional and with TiDi-Fused pipelines. All white matter regions of interest had lower CoV measurements in the tracts derived from the TiDi-Fused pipeline compared to those calculated from images processed with the conventional pipeline (Figure 4A). A Wilcoxon signed-rank exact test comparing the CoVs of brainstem regions processed with the conventional (*M* = 9.3%, SD = 1.8%) and TiDi-Fused pipelines (*M* = 7.2%, SD = 1.6%) showed that CoV was significantly reduced in the TiDi-Fused pipeline, *z* = −5.15, *p* < 2.4e-07. Across the regions, the reduction in CV was on average 2.1% (95% confidence interval: 1.7% to 2.5%).

In 21 of 23 white matter tracts, the AFD values extracted from TiDi-Fused processed data showed stronger correlations with age, as indexed by higher R^2^, than those extracted from the conventional pipeline (Figure 4B, Supplementary Table 1). The only tracts that did not show improved linear fit were in the left inferior cerebellar peduncle (ICPMC and ICPVC). Further, a positive relationship between AFD and age was found in 23 of 23 tracts using the TiDi pipeline but only 12/23 tracts using the conventional pipeline (Supplementary Table 2).

Linear mixed effects models were used to determine whether the dMRI pipeline optimization significantly impacted estimates of the relationship between age and AFD. In 21 of 23 brainstem white matter tracts, significant age-by-pipeline interactions (*p* < 0.05) were found (Supplementary Table 3), indicating that the optimized dMRI pipeline statistically altered estimates of AFD-age relationships. The left SCPSC and left frontopontine tracts were the only white matter regions not to show statistically significant age-by-pipeline interactions.

## Discussion

To set the stage for *in vivo* testing of hypotheses regarding brainstem contributions to autism symptoms, this study set out to implement and test a T1w-dMRI fused (TiDi-Fused) processing pipeline that enhances resolution and accurate delineation of brainstem structures in autistic and non-autistic children. The TiDi-Fused pipeline harnesses the strengths of T1w and dMRI imaging techniques to generate high apparent resolution dMRI maps without sacrificing SNR or requiring long scan times. Previously, high brainstem anatomical clarity using dMRI has only been possible through the use of *ex vivo* imaging (Ford et al., 2013) or lengthy acquisition protocols (~60 min) that are not suitable for a pediatric population (Shi and Toga, 2017), making the present application of TiDi-Fusion to a neurodiverse pediatric sample a critical advancement in pediatric neuroimaging. Through direct comparison of our TiDi-Fused pipeline and our conventional dMRI processing pipeline, we demonstrated substantial improvement in visualization, alignment, and quantification of brainstem structures in autistic and non-autistic youth.

TiDi-Fused processing greatly improved the ability to distinguish white matter tracts and gray matter nuclei in the anatomically complex brainstem, leading to high-resolution representations of brainstem structures in autistic children from data collected *in vivo*. TiDi-Fused processing enhanced visual assessment of brainstem white matter pathways and improved alignment with a histological atlas of precisely delineated gray matter nuclei. These high-resolution brainstem images and well-defined regions of interest generated from the TiDi-Fused processing pipeline provide the opportunity to test hypotheses regarding the contributions of the brainstem to autism that have been produced in other scientific fields [e.g., histology, cellular biology, model organisms; reviewed by Dadalko and Travers (2018)]. For example, TiDi-Fused processing offers the opportunity to delineate brainstem structures, like the reticular formation, which was at the heart of Rimland’s (1964) hypothesis. In this way, TiDi-Fused processing may now enable examinations into the relatively unexplored territory of the brainstem in autism and in other difficult-to-image populations with conditions that may involve the brainstem (e.g., Alzheimer’s or Parkinson’s disease; Halliday et al., 1990; Simic et al., 2009; Arribarat et al., 2020). Moreover, while the TiDi-Fused pipeline involves registration with the T1w component of the MPnRAGE sequence, future work may benefit from fusing the dMRI with other quantitative structural MRI images or contrasts, which may provide additional information about brainstem composition.

Compared to the conventional pipeline, the TiDi-Fused pipeline not only enhanced the quality of brainstem visualization but also statistically improved the reliability of microstructural property measurements. This quantitative improvement of the TiDi-Fused pipeline over the conventional pipeline was demonstrated in two ways: (1) lower variability (CoV) across all measurements of AFD in brainstem white matter tracts; and (2) stronger relationships with age. The increased reliability of the TiDi-Fused data, as indexed by lower CoV across all brainstem white matter tracts, is especially critical in the brainstem, as the brainstem white matter bundles are smaller in size than most cerebral white matter structures and can be heavily impacted by spurious measurements. This notion is further supported by the improved estimates of AFD-age relationships in the data processed with the TiDi-Fused pipeline compared to the data processed with the conventional pipeline. AFD values from TiDi-Fused data also show more biologically plausible (positive) age trajectories that are corroborated by previous accounts of AFD development of large white matter tracts in non-autistic youth (Dimond et al., 2020) and align with previous work done to assess diagnostic differences in white matter development in various white matter tracts throughout the brain (Andrews et al., 2021). The TiDi-Fused processing pipeline, therefore, appears to allow for more reliable estimates of white matter microstructure (less variability) and improved biological plausibility, which can, in turn, enhance the validity of *in vivo* assessments of brainstem-behavior relationships in autistic and non-autistic youth.

The present findings should be contextualized in light of limitations. One potential limitation was that we compared across different apparent resolutions: 1.3 mm^3^ in the conventional scans compared to 1.0 mm^3^ in the TiDi-Fused scans. While a more direct comparison would have been to compare 1 mm^3^ to 1 mm^3^, we opted to keep our conventional scan at the MRTrix3 recommended apparent resolution of 1.3 mm^3^ (Fibre density and cross-section - Multi-tissue CSD) to maintain consistency with current best practices. Another limitation is that we did not have a measure of ground truth for brainstem neurobiology in our participants. To compensate, we opted for visual alignment with histology data and quantitative measures that examined reliability (CoV) and biological plausibility (age effects). However, future work should examine additional measures to validate this approach.

Overall, the TiDi-Fused processing pipeline demonstrated enhanced assessment of brainstem structures in autistic children, providing the opportunity to conduct feasible *in vivo* investigations of the brainstem that has not to date been possible. The TiDi-Fused processing pipeline increases apparent spatial resolution without compromising SNR or requiring long scan times, resulting in both visual and quantitative improvements to brainstem analysis in autistic and non-autistic children. Therefore, the present pipeline represents a critical advancement in our ability to use MRI to understand the role of the brainstem in autism.

## Data Availability Statement

The raw data supporting the conclusions of this article will be made available by the authors, without undue reservation.

## Ethics Statement

The studies involving human participants were reviewed and approved by University of Wisconsin-Madison Health Sciences Institutional Review Board. Written informed consent to participate in this study was provided by the participants’ legal guardian/next of kin.

## Author Contributions

JG-G, OS, NA, GK, DD, AA, and BT contributed to conception and design of the study. JG-G, OS, NA, DD, SK, and AA made contributions to image processing. NA and OS performed the statistical analysis. OS wrote the first draft of the manuscript. JG-G, NA, and BT wrote sections of the manuscript. All authors contributed to the article and approved the submitted version.

## Conflict of Interest

AA is part owner of ImgGyd, LLC and inseRT MRI, Inc. (also listed as TherVoyant). While both companies are involved in developing MRI-based surgery techniques, neither are associated with any current areas of his research, including the present publication. All other authors report no biomedical financial interests of potential conflicts of interest. The remaining authors declare that the research was conducted in the absence of any commercial or financial relationships that could be construed as a potential conflict of interest.

## Publisher’s Note

All claims expressed in this article are solely those of the authors and do not necessarily represent those of their affiliated organizations, or those of the publisher, the editors and the reviewers. Any product that may be evaluated in this article, or claim that may be made by its manufacturer, is not guaranteed or endorsed by the publisher.

## Footnotes

^1^Identity-first language is used throughout the article to reflect the preference of those in the autism community (Kenny et al., 2016; Bottema-Beutel et al., 2021).

^2^ https://mrtrix.readthedocs.io/en/3.0.3/fixel_based_analysis/mt_fibre_density_crosssection.html

^3^ http://www.bic.mni.mcgill.ca/ServicesAtlases/ICBM152NLin2009
