# Supplementary material for: Improving Imaging of the Brainstem and Cerebellum in Autistic Children: Transformation-Based High-Resolution Diffusion MRI (TiDi-Fused) in the Human Brainstem
Source: Front Integr Neurosci. 2022 Mar 3;16:804743. doi: 10.3389/fnint.2022.804743 (PMC8928227; doi:10.3389/fnint.2022.804743)
Supplement: Supplementary file 1 [file Data_Sheet_1.PDF]

Supplementary Material

1 Supplementary Data

FOD creation

Three study specific templates were generated using FODs derived from the three tissue (white matter, gray matter and CSF) multi-shell response functions. An image based on the combined mean squared amplitude of the three FOD templates was then generated to register the brainstem atlas which was in MNI-152 T1w space. The MNI-152 T1w template was registered to the mean squared amplitude image using Advanced Normalization Tools (ANTs) with presets shown in detail in the code snippet below. These estimate translation, rigid, affine and SyN transformations using a combination of mutual information (MI), cross-correlation (CC) and global correlation (GC) metrics. The composite warps were then applied to the 23 bundles using linear interpolation to transform them to the FOD template space. These bundles were then warped with cubic interpolation into the subject specific native space using the inverse of the warps estimated when generating the FOD templates.

Code Snippet:

```
fI=HM_FOD_Overall_P_LONIMO.nii.gz
mI=MNI152_T1_SS_RAS_1mm.nii.gz
antsRegistration -d 3 --float 1 --verbose 1 -u 1 -w [0.001, 0.999] -z 1 \
\
-r [$fI, $mI, 1] \
-t Translation[0.1] \
-m MI[$fI, $mI, 1, 32, Regular, 0.25] -c [1000x500x250x50, 1e-7, 10] -f 8x4x4x1 -s 5x4x3x1vox -l 1\
-t Translation[0.1] \
-m MI[$fI, $mI, 1, 32, Regular, 0.25] -c [1000x500x250x50, 1e-7, 10] -f 4x4x4x1 -s 2x2x1x1vox -l 1\
-t Translation[0.1] \
-m CC[$fI, $mI, 1, 4] -c [1000x500x250x50, 1e-7, 10] -f 8x4x4x1 -s 4x3x1x1vox -l 1 \
-t Translation[0.1] \
-m CC[$fI, $mI, 1, 4] -c [1000x500x250x50, 1e-7, 10] -f 4x4x4x1 -s 2x2x1x1vox -l 1 \
-t Translation[0.1] \
-m GC[$fI, $mI, 1, 15, Regular, 0.05] -c [1000x500x250x50, 1e-7, 10] -f 8x4x4x1 -s 4x3x1x1vox -l 1\
-t Translation[0.1] \
-m GC[$fI, $mI, 1, 15, Regular, 0.05] -c [1000x500x250x50, 1e-7, 10] -f 4x4x4x1 -s 2x2x1x1vox -l 1\
-t Rigid[0.1] \
-m MI[$fI, $mI, 1, 32, Regular, 0.25] -c [1000x500x250x50, 1e-7, 10] -f 8x4x4x1 -s 5x4x3x1vox -l 1\
-t Rigid[0.1] \
-m MI[$fI, $mI, 1, 32, Regular, 0.25] -c [1000x500x250x50, 1e-7, 10] -f 4x4x4x1 -s 2x2x1x1vox -l 1\
-t Rigid[0.1] \
-m CC[$fI, $mI, 1, 4] -c [1000x500x250x50, 1e-7, 10] -f 8x4x4x1 -s 4x3x1x1vox -l 1 \
-t Rigid[0.1] \
-m CC[$fI, $mI, 1, 4] -c [1000x500x250x50, 1e-7, 10] -f 4x4x4x1 -s 2x2x1x1vox -l 1 \
-t Rigid[0.1] \
-m GC[$fI, $mI, 1, 15, Regular, 0.05] -c [1000x500x250x50, 1e-7, 10] -f 8x4x4x1 -s 4x3x1x1vox -l 1\
-t Rigid[0.1] \
-m GC[$fI, $mI, 1, 15, Regular, 0.05] -c [1000x500x250x50, 1e-7, 10] -f 4x4x4x1 -s 2x2x1x1vox -l 1\
\
-t Affine[0.1] \
-m MI[$fI, $mI, 1, 32, Regular, 0.25] -c [1000x500x250x50, 1e-7, 10] -f 8x4x4x1 -s 4x3x1x1vox -l 1\
-t Affine[0.1] \
-m MI[$fI, $mI, 1, 32, Regular, 0.25] -c [1000x500x250x50, 1e-7, 10] -f 4x4x4x1 -s 2x2x1x1vox -l 1\
-t Affine[0.1] \
-m CC[$fI, $mI, 1, 4] -c [1000x500x250x50, 1e-7, 10] -f 8x4x4x1 -s 4x3x1x1vox -l 1 \
-t Affine[0.1] \
-m CC[$fI, $mI, 1, 4] -c [1000x500x250x50, 1e-7, 10] -f 4x4x4x1 -s 2x2x1x1vox -l 1 \
-t Affine[0.1] \
-m GC[$fI, $mI, 1, 15, Regular, 0.05] -c [1000x500x250x50, 1e-7, 10] -f 8x4x4x1 -s 4x3x1x1vox -l 1\
-t Affine[0.1] \
-m GC[$fI, $mI, 1, 15, Regular, 0.05] -c [1000x500x250x50, 1e-7, 10] -f 4x4x4x1 -s 2x2x1x1vox -l 1\
\
-t SyN[0.3, 4, 3] \
-m MI[$fI, $mI, 1, 32,Regular,0.25] -c [1000x500x500x100, 1e-9, 10] -f 8x4x4x1 -s 4x3x1x1vox -l 1 \
```

```
-t SyN[0.3, 4, 3] \
-m MI[$fI, $mI, 1, 32,Regular,0.25] -c [200x50x10x10, 1e-9, 10] -f 4x4x4x1 -s 2x2x1x1vox -l 1 \
-t SyN[0.3, 4, 3] \
-m CC[$fI, $mI, 1, 4] -c [1000x500x500x100, 1e-9, 10] -f 8x4x4x1 -s 4x3x1x1vox -l 1 \
-t SyN[0.3, 4, 3] \
-m CC[$fI, $mI, 1, 4] -c [200x50x10x10, 1e-9, 10] -f 4x4x4x1 -s 2x2x1x1vox -l 1 \
-t SyN[0.3, 4, 3] \
-m GC[$fI, $mI, 1, 15, Regular, 0.05] -c [1000x500x500x100, 1e-9, 10] -f 8x4x4x1 -s 4x3x1x1vox -l 1 \
-t SyN[0.3, 4, 3] \
-m GC[$fI, $mI, 1, 15, Regular, 0.05] -c [200x50x10x10, 1e-9, 10] -f 4x4x4x1 -s 2x2x1x1vox -l 1 \
-o loni_overall_p
```

2 Supplementary Figures and Tables

2.1 Supplementary Figures

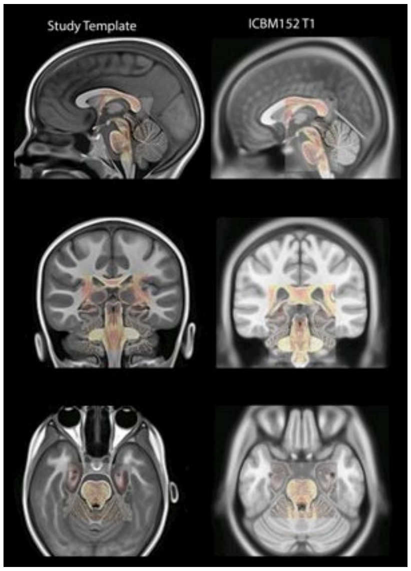

Supplementary Figure 1. BigBrain histology in ICBM152 space according to Sitek et al. 2009 (right side column) and aligned to the study specific T1-weighted template (left side column).

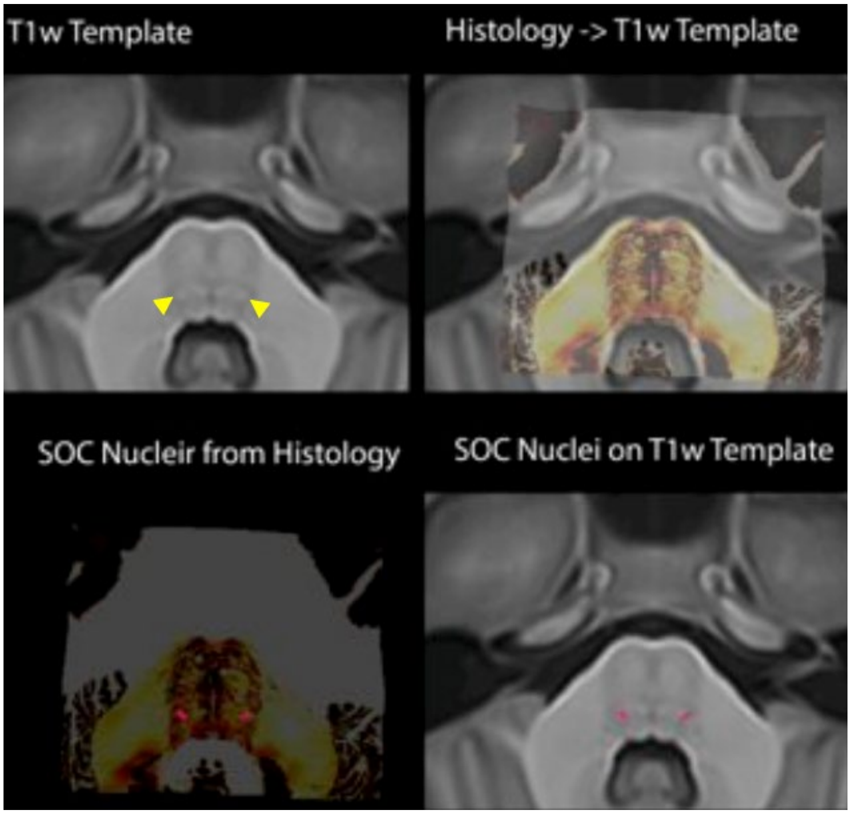

Supplementary Figure 2. Upper left corner shows a region in the brainstem on the study specific template where some structure is visible (yellow arrowheads). The upper right corner shows the histology contrast overlaid on the same region the the upper left panel after alignment to the study specific template. In the lower left corner a portion of the superior olivary complex is shown delineated on the histology by Sitek et. al. 2019. The lower right corner shows this regions overlapping with the contrast observed in the study specific template.

2.2 Supplementary Tables

Supplementary Table 1. Demographic information for participant sample

	Autistic (n=56)	Non-Autistic (n=72)
Age(years), Mean(SD)	8.53(1.33)	8.31(1.40)
Sex, % Female	22%	35%
Average Head Motion (RMS), Mean(SD)	0.53(0.40)	0.47(0.40)
IQ, Standardized Score, Mean(SD)	103(22)	114(12)
SCQ, Mean(SD)	19.71(7.02)	1.46(1.87)

RMS Root Mean Squared; SCQ Social Communication Questionnaire; Standardized IQ scores are harmonized across participants who completed the Kaufman Brief Intelligence Test, Second Edition (KBIT-2; Autistic n=10, Non-Autistic n=22) or the Wechsler Abbreviated Scale of Intelligence, Second Edition (WASI-II; Autistic n=46, Non-Autistic n=50)

Supplementary Table 2. Correlations between AFD and age in brainstem white matter regions of interest

Brainstem White Matter Region	Conventional Pipeline r(p)	TiDi-Fused Pipeline r(p)
CSTL	+.27(.01)	+.53(<.001)
CSTR	+.32(.002)	+.50(<.001)
MLL	+.07(.53)	+.44(<.001)
MLR	+.01(.94)	+.40(<.001)
LLL	-.03(.74)	+.23(.03)
LLR	+.01(.96)	+.34(.001)
FPTL	+.14(.19)	+.26(.01)
FPTR	+.15(.16)	+.28(.008)
POTPTL	+.05(.64)	+.44(<.001)
POTPTR	+.08(.42)	+.54(<.001)
STTL	+.05(.64)	+.33(.001)
STTR	+.08(.81)	+.41(<.001)
SCPCTL	+.05(.27)	+.28(.007)
SCPCTR	-.02(.27)	+.31(.003)
SCPCRL	-.12(.24)	+.21(.05)
SCPCRR	-.12(.25)	+.20(.06)
SCPSCL	-.12(.37)	+.28(.008)
SCPSCR	+.09(.79)	+.29(.005)
MCP	+.03(.13)	+.22(.04)
ICPMCL	-.16(.11)	0.13(.25)
ICPMCR	-.17(.37)	0.21(.05)
ICPVCL	-.10(.10)	0.13(.20)
ICPVCR	-.10(.34)	0.17(.10)

Partial correlations account for age, sex and average head motion during DWI brain scan
ML medial lemniscus; LL lateral lemniscus; STT spinothalamic tract; SCPCT superior cerebellar peduncle cerebellothalamic tract; SCPSC superior cerebellar peduncle spinocerebellar tract; MCP middle cerebellar peduncle; ICPMC inferior cerebellar peduncle tracts from medulla oblongata to the cerebellum; ICPVCT inferior cerebellar peduncle vestibulocerebellar tract; FPT frontopontine tract; POTPT parieto-occipito-temporo-pontine tract; CST corticospinal tract

Supplementary Table 3. Age-by-pipeline interaction terms from mixed effects linear models predicting AFD in brainstem white matter regions of interest

Brainstem White Matter Region	b	SE	*t*	*p*
CSTL	0.005	0.002	3.03	0.002
CSTR	0.006	0.002	3.06	0.002
MLL	0.007	0.002	3.55	<.001
MLR	0.007	0.002	3.37	<.001
LLL	0.011	0.003	4.05	<.001
LLR	0.01	0.003	3.80	<.001
FPTL	0.003	0.002	1.85	0.06
FPTR	0.005	0.002	2.17	0.03
POTPTL	0.008	0.002	3.97	<.001
POTPTR	0.01	0.002	4.76	<.001
STTL	0.006	0.002	3.61	<.001
STTR	0.01	0.002	4.86	<.001
SCPCTL	0.007	0.002	4.023	<.001
SCPCTR	0.007	0.002	4.22	<.001
SCPCRL	0.007	0.002	3.79	<.001
SCPCRR	0.008	0.002	4.19	<.001
SCPSCL	0.003	0.002	1.49	0.13
SCPSCR	0.006	0.002	3.57	<.001
MCP	0.007	0.001	4.24	<.001
ICPMCL	0.008	0.002	3.42	<.001
ICPMCR	0.008	0.002	3.31	0.001
ICPVCL	0.009	0.002	3.51	<.001
ICPVCR	0.008	0.003	3.21	0.002

ML medial lemniscus; LL lateral lemniscus; STT spinothalamic tract; SCPCT superior cerebellar peduncle cerebellothalamic tract; SCPSC superior cerebellar peduncle spinocerebellar tract; MCP middle cerebellar peduncle; ICPMC inferior cerebellar peduncle tracts from medulla oblongata to the cerebellum; ICPVCT inferior cerebellar peduncle vestibulocerebellar tract; FPT frontopontine tract; POTPT parieto-occipito-temporo-pontine tract; CST corticospinal tract
